# Structural assessment of the gripper interlock of the DEMO breeding blanket transporter

**DOI:** 10.1016/j.heliyon.2023.e18926

**Published:** 2023-08-03

**Authors:** Rocco Mozzillo, Christian Bachmann, Pierluigi Fanelli, Guenter Janeschitz, Thomas Steinbacher

**Affiliations:** a CREATE, Engineering School of Basilicata University, Campus Macchia Romana, PZ, 85100, Italy; b EUROfusion Consortium, FTD Department, Garching, Boltzmannstr. 2, Germany; c Department of Economics, Engineering, Society and Business Organization, University of Tuscia, Largo dell’Università, 01100, Viterbo, Italy; d Max-Planck-Institut für Plasmaphysik, 85748, Garching, Germany

**Keywords:** DEMO, Remote maintenance, Breeding blanket transporter, Gripper interlock

## Abstract

The maintenance of the DEMO Breeding Blanket (BB) remotely is a crucial aspect in development of the DEMO power plant. It is a challenge due to the huge mass of the BB segment of about 180 tons. A new concept for the BB transporter has recently been developed. To properly grip and manipulate each BB segment, the BB transporter has been equipped with a gripper interlock. Due to the geometry of the BB and the vacuum vessel, the attachment point on the BB segment is not aligned with its center of gravity. Hence in addition to the vertical lifting load, large moments about the horizontal axes need to be reacted.

The work discussed here concerns the structural analysis conducted on the gripper interlock; its structural integrity has been checked against the most severe load conditions that include also seismic loads according to the EN13001. Elastic analyses were performed using a finite element model in accordance with EN 13001-3-1:2012 + A2:2018, Cranes - General Design - Part 3–1: Limit States and proof competence of steel structure. The effect of the gap sizes at the contact surfaces between gripper interlock and BB after engagement as well as the effect of different friction coefficients on the sliding areas were assessed. The improvements of the design based on the structural analysis are presented, too.

## 1 Introduction

DEMO could be one of the next step towards the design and development of commercial fusion power plants. Its main aims consist of demonstrating the production of few hundred MW of net electricity, the feasibility of operation with a closed-tritium fuel cycle, and a maintenance system capable of achieving adequate plant availability [1]. Tritium is assumed as produced inside a breeding blanket (BB) covering the internal wall of the vacuum vessel (VV). The degradation of the materials of these critical components requires regular replacement [2] and the remote maintenance processes must be tested to be consequently adopted in a fusion power. Due to this the BB maintenance strategy consists in increasing as much as possible the power plant availability, on one hand, and on the other in reducing the maintenance costs. The remote replacement of the BB was identified one of the crucial aspects of the EU DEMO [3] development. Recently a new approach of BB maintenance strategy has been proposed [4]. As in ITER, a sealed environment is adopted to carry out remote replacement of the In- Vessel Components (IVCs) [5,6]. A proper cask, sealed to the VV is designed to assure a double containment during the remote handling operations.

Accordingly, an innovative design for the BB transporter has been proposed too [4]. The BB is removed and re-installed from within a containment box on top of the bioshield roof through the upper ports of the VV. Separate containment boxes placed on top of each upper port guarantee that in-vessel operations can be potentially carried out in all upper ports independently. Due to the presence of the magnetic coils the VV access ports are limited in space and they do not allow for direct lifting of the BB segments [7]. In addition, tilting movements of the BB segments are also needed to disengage the BB segments from their supports and to move them through the upper port clear of the in-situ BB segments partially obstruct the upper port [8]. The BB transporter is equipped with a trunk that can rotate about the vertical axis; at its end the gripper interlock (GI) is mounted. The main function of this component is the engagement with the BB segments. Due to constrained accessibility of the backside of some of the BB segments, the GI is the highest loaded part of the entire transporter. It is subjected to high moments about the toroidal and radial axes of the machine. The most critical condition occurs during the initial lifting of the inboard segments. The design of the GI itself is described in Ref. [9]. This article addresses (i) the verification of its structural integrity against the most severe load condition, (ii) an assessment of the gap size on the contact surfaces with BB segment, and (iii) an evaluation of the impact different friction coefficients on the stress distribution on the GI structure.

## 2 Current design

The Gripper Interlock (GI) has been designed to transfer loads (mainly bending moments) across the small accessible surface placed on the backside of the BB segments. The lifting surfaces of lateral outboard segments are particularly narrow. The cross-section of the gripper interlock was chosen rectangular with a shorter toroidal and a longer radial edge reflecting the accessible surface on the BB segments.

**Fig. 1:**
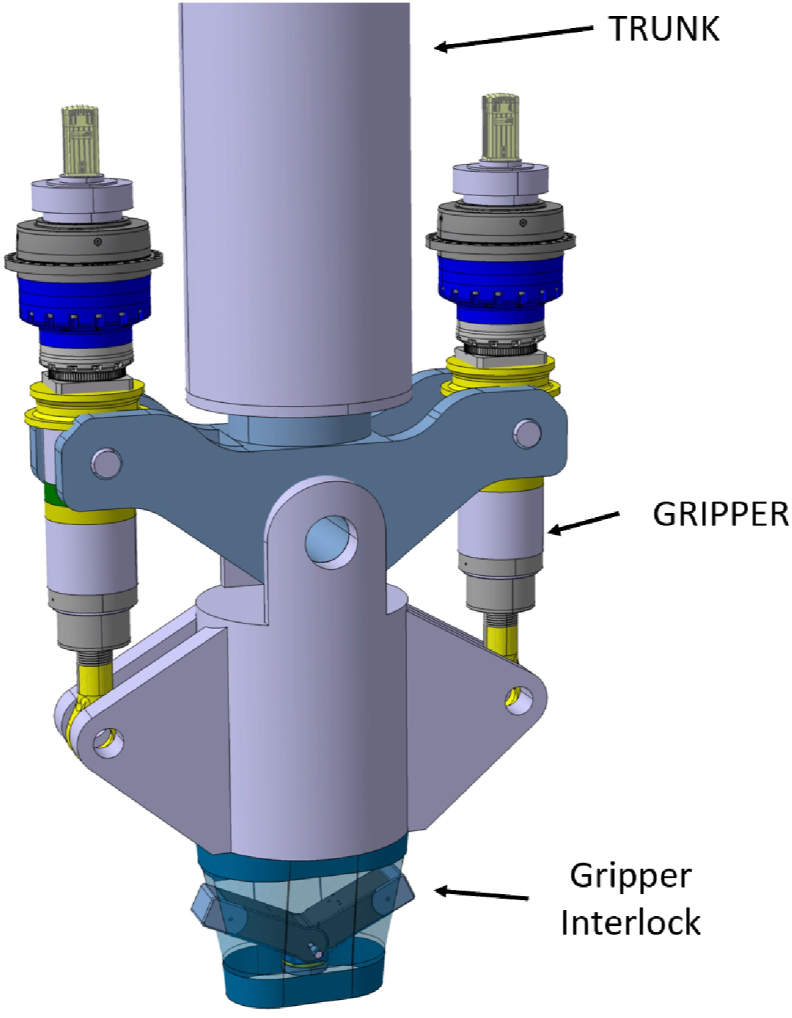
Overview of current design of trunk, gripper, and gripper interlock.

The GI is mounted on the extremity of the trunk (Fig. 1) of BB transporter [10]. The lower part of the gripper, the GI, is attached to the gripper mounting shaft by large pivot pins on both sides, which define the center of the tilting motion. These pins transfer the vertical and horizontal forces as well as the bending moment about the second horizontal axis. The bending moment about the tilting axis is borne by the actuators (Fig. 1).

**Fig. 2:**
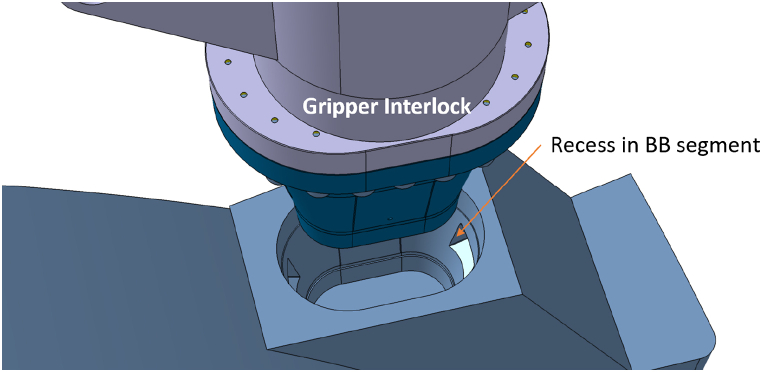
Recess in BB segment.

The GI shall guarantee the lifting of each BB segment and withstand the safety removal of the BB segment. The GI has been designed also for an SL-1 Seismic event [11,12] since the remote handling operations can have relevant duration. The GI is entered into a recess in the backside of the BB segment chimney and mechanically locks itself with the BB segment (Fig. 2).

**Fig. 3:**
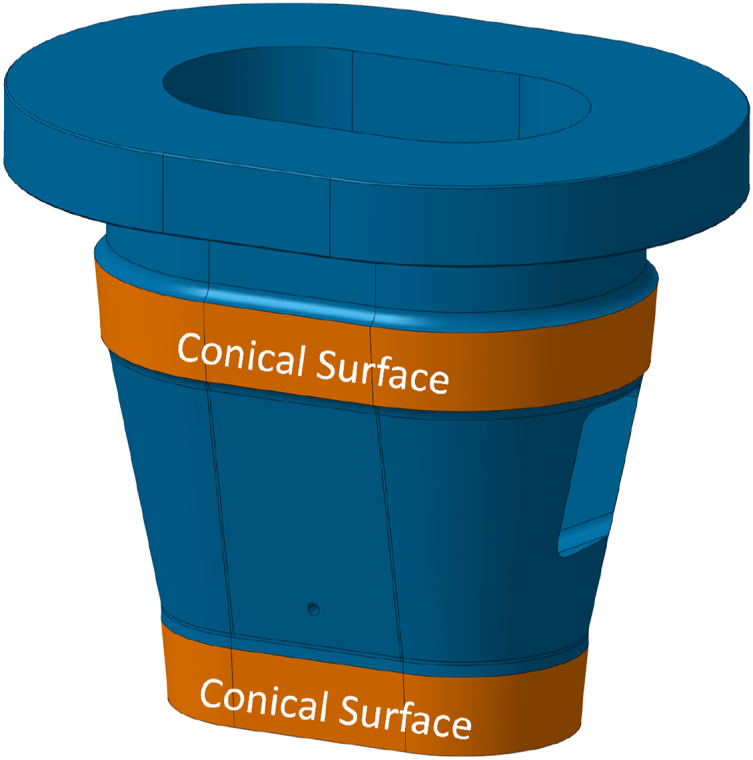
Conical surfaces on the housing to transfer horizontal forces reacting bending moments.

To enable guided engagement, it has a somewhat conical shape (Fig. 3).

**Fig. 4:**
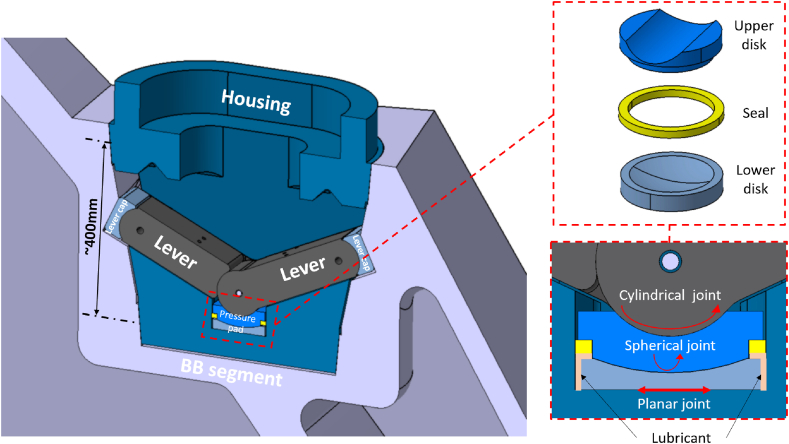
Functional scheme (section on midplane of the GI).

**Fig. 5:**
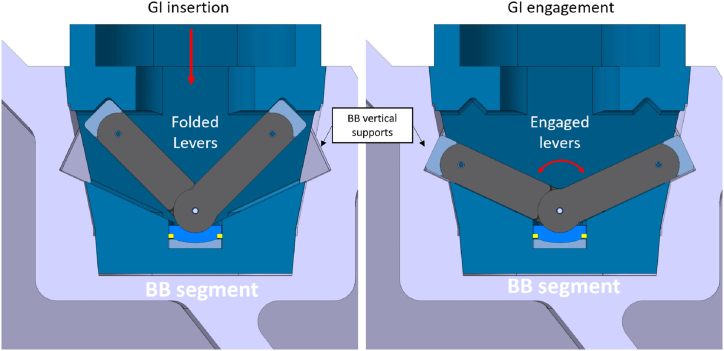
GI insertion and engagement (Section on mid-plane of GI).

The GI is composed of a housing structure joined to the gripper by a bolted flange [9], The vertical loads are supposed to be reacted by the levers inclined by an angle of 65° with respect to the vertical axis in the engaged configuration (Fig. 4). During the insertion of the GI into the BB recess, the levers are folded. The levers are then unfolded and engage with the contact areas on the BB upon lifting (Fig. 5). The locking levers are equipped with lever caps with cylindrical joints.

The pressure pad, placed on the bottom area of the GI, transfers the vertical loads from the two levers to the housing and the rest of BB transporter structure. It can slide on the horizontal plane (toroidal-radial plane) by means of a planar join. The pressure pad is composed by an upper disk interfacing on top to the cylindrical surfaces of the levers, and on the bottom to the spherical surface of lower disk. The lower disk interacts with the housing through a planar surface on the bottom (Fig. 4). The main function of the pressure pad consists in transferring only the vertical loads from the levers to the housing and reducing as much as possible the stress flowing into the levers due to the moments. This area has been assumed as lubricated and a seal has been implemented to avoid lubricant leakage. Thus, a friction coefficient of 4% has been assumed on the spherical and the planar joints (Fig. 4).

## 3 Structural analyses

The design loads considered in the structural integrity verification are shown in Ref. [10]. Dead weight (DW) and seismic acceleration due to an SL-1 event are considered. The assumed lifted masses according to Ref. [4] are 125 tons for the inboard segments and 180 tons for outboard segments. An SL-1 seismic event has been considered with maximum equivalent accelerations of the BB segments of ∼0.8 m/s^2^ and ∼2.7 m/s^2^ in horizontal and vertical directions, respectively. The design loads were defined considering to the partial safety factors defined in EN 13001 [13] i.e., 1.41 DW + 1.1 SL-1.

Our study concerned not only the structural verification of the GI itself but also the effect of the friction coefficients between GI components and the assembly gaps after GI engagement.

Provisionally ultra-high-strength S960 steel (EN 10025–6) [14,15] was chosen as material for the GI components with a minimum yield stress of f_y_ = 960 MPa. EUROFER has been considered as material for the BB segment [16].

The loads were applied in two subsequent load steps: 1) vertical load, 2) vertical load + bending moments. The rules for elastic analysis defined in EN13001 were considered, which defines the allowable stress as 752 MPa for rolled steel (f_y_/(1.1 × 1.16) - for material in quality class Z15 in accordance with EN 10164:2004 and as 1040 MPa (f_y_/(1.1 × 0.95)) for non-rolled material. The housing is assumed to be manufactured as cast steel and machined while the others components are made from rolled material.

### 3.1 Structural integrity verification

**Fig. 6:**
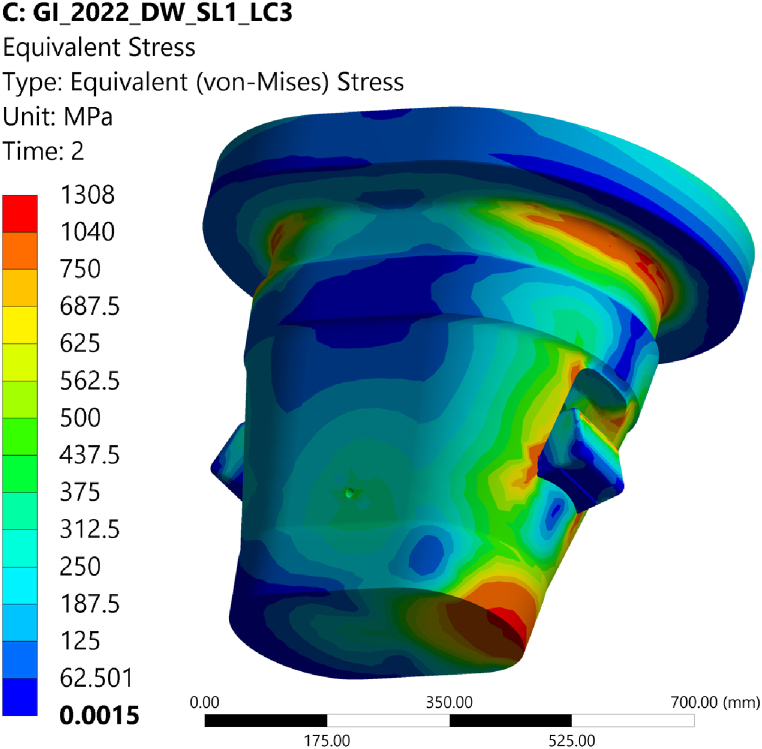
Equivalent von Mises stress distribution in the GI – lifting of the BB inboard segments, red areas indicate stress levels above the allowable.

A linear elastic analysis was carried out considering the worst case loading condition (see Table 2): lifting of the inboard segment, see Table 1. The nominal size of the engagement gap on the conical contact surfaces of 0.5 mm was assumed [9]. The stress level in the fillet of the upper flange, on the edge of the lower conical contact surface and in the corner of the cut-out in the housing is near the allowable stress and it suggests small improvement in design of these areas, an option to reduce the level of the stress could be to increase the fillet radii (Fig. 6). Since these are local regions of the structure, we expected these were not membrane but rather bending and peak stresses. To verify this assumption a subsequent limit analysis was carried out.

**Table 1:** Interface design loads transferred from the BB segments to the GI due to dead weight and seismic event i.e., 1.41 DW + 1.1 SL-1 [17].

BB segment	F_vert_	M_tor_	M_rad_
Inboard	2.2 MN	4.4 MNm	0.6 MNm
Lateral outboard	3.1 MN	0.8 MNm	1.4 MNm

**Table 2:** Contact characteristics of the GI joints.

Interfaces	Contact	Friction coefficient
Levers caps vs. BB	Dry	30%
Lever cap vs. lever	Dry	4, 15, 30%
Right lever vs. left lever		
Levers vs. pressure pad		
Pressure pad upper disk vs. lower disk	Lubricated	4%
Pressure pad lower disk vs. housing

**Fig. 7:**
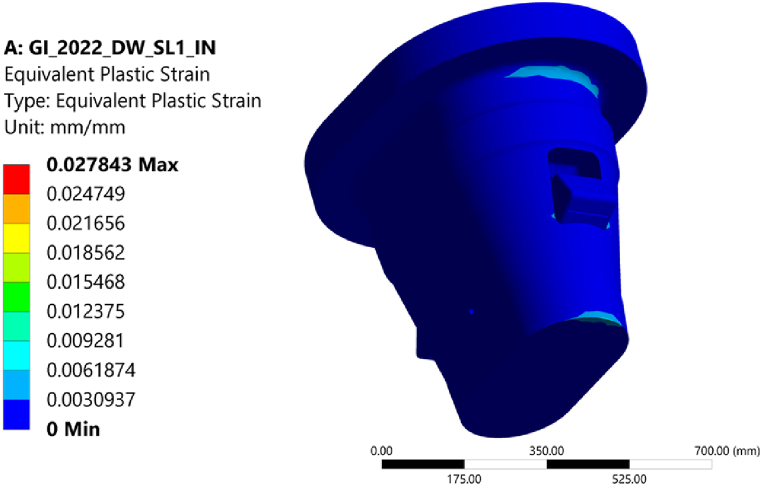
Equivalent plastic strain at load factor 1.5.

A limit analysis was carried out to distinguish membrane from bending and peak stresses in the three-dimensional structure of the GI. In absence of a reference limit for the collapse load factor in EN13001 [13] RCC-MRx [18] rules were adopted. A linear elastic perfectly plastic material behavior was defined, and the load was increased progressively up to the collapse of the structure, applying it to the undeformed geometry [19]. The collapse of the structure was found to occur at a load factor of 3.2, well above the required load factor of 1.5, as requested by RB 3251.112 [18]. This verifies that the surrounding structure has sufficient reserve to prevent excessive deformation failure. At a load factor of 1.5 the plastic strain is limited to no more than 1% (Fig. 7).

### 3.2 Engagement gap size assessment

The assessment of the gap size between the GI working surfaces and the corresponding on the BB recess has been conducted in two different steps: in the first the gaps between the working surfaces of the vertical supports (i.e. lever cap top surfaces on the corresponding on the BB recess), the second concerned the gaps between the conical surfaces of the housing and the corresponding on the BB recess.

**Fig. 8:**
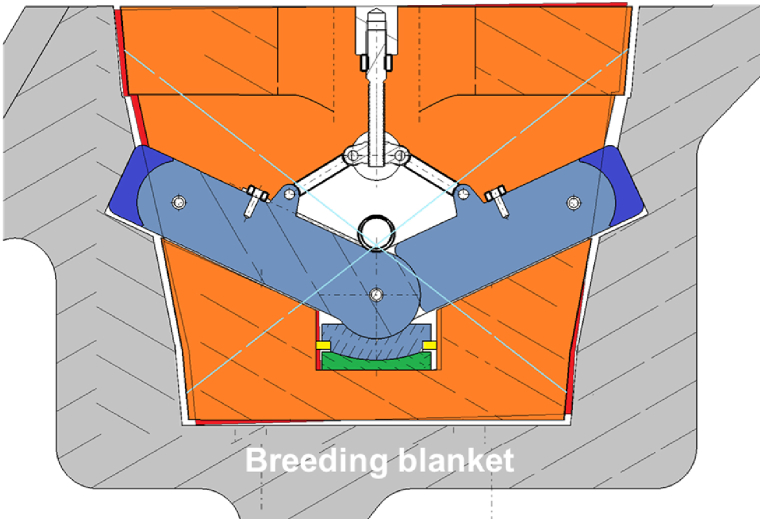
Gap configurations - **red contou**r Gripper Interlock during lifting, gaps on the horizontal support surfaces closed **– blue contour** Vertical support engaged - gaps between the lever caps surfaces closed.

The analyses included an assessment on the gaps allowed on the working surfaces (Fig. 8). The outcomes of the analyses give information about the allowable assembly tolerances of the GI and surface finishing of the surfaces of the BB recess. The assessment has been conducted assuming gaps sizes varying in the range 0–3 mm with steps of 0.5 mm both on the toroido-radial supports (conical surfaces of the GI housing and the corresponding on the BB recess) and on the vertical supports (top surfaces of the lever cap and the corresponding on the BB recess).

**Fig. 9:**
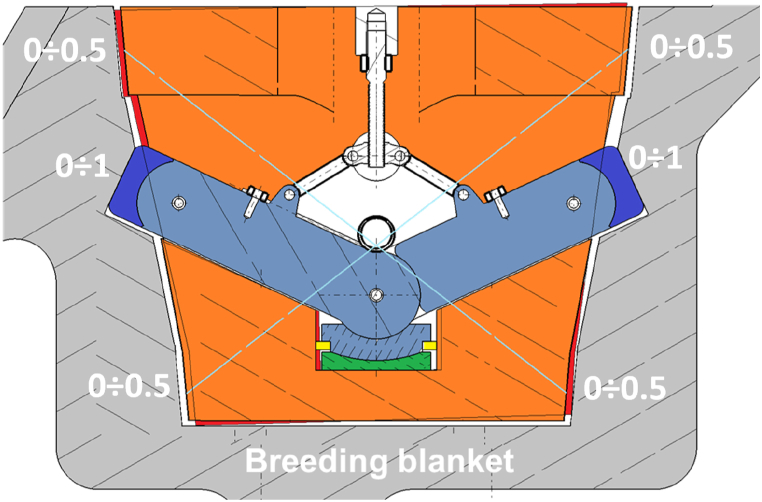
Gap ranges layout – gap sizes in mm.

Results of the analyses showed for gap sizes lower than 1 mm between the lever arm caps and the BB and gap sizes lower than 0.5 mm between the conical surfaces of the GI housing and the corresponding on the BB recess the GI engages well with the BB (Fig. 9) and the stress level is similar to the configuration with 0 mm of gap.

**Fig. 10:**
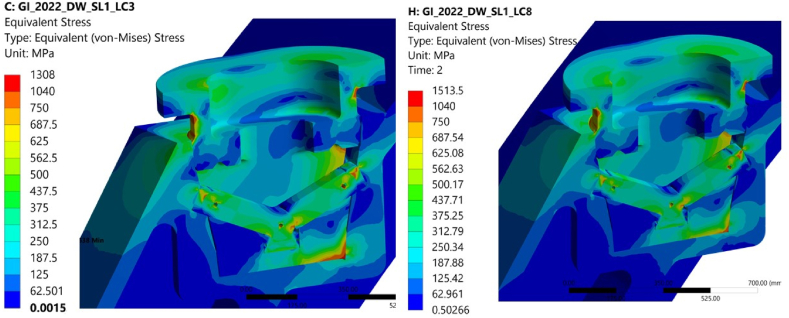
Equivalent Von Mises Stress - **Left image** 0.5 mm of gap on the conical surfaces; **right image** 0 mm of gap on the conical surfaces - Other parameters (gaps, friction, coefficients on the other surfaces) are the same in both configuration.

**Fig. 11:**
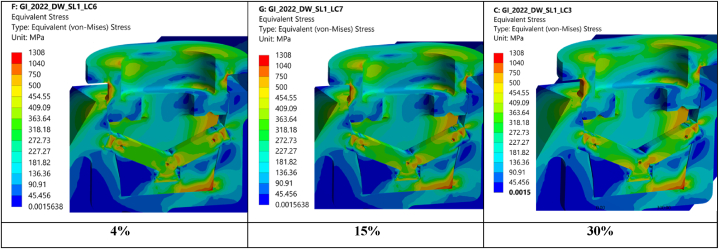
Equivalent von Mises stress in GI due to worst case loading condition for different values of the postulated friction coefficient on the dry contact surfaces – **4% left, 15% central, 30% right**.

The scope of the work consisted in definition of these limits and checking the level of the stress mainly on the weak component of the GI (i.e. lever and lever cap) due to un-symmetrical load conditions caused by geometrical tolerances. The stress level in each configuration has been also addressed, some of the results are shown in Fig. 11. In all configuration the areas subjected to high level of Von Mises Stress are the once highlighted in chapter 3.1 and in Fig. 6. Fig. 10 shows the GI can tolerate gap of 0.5 mm on the conical surfaces, in the image on the left the stress level is similar to the case with 0 mm of gap on the conical surfaces of the GI.

### 3.3 Impact of different friction coefficients on the sliding areas between GI and BB segment

To assess the effect of the friction coefficient on the dry contact surfaces a set of FEM analyses was run with different postulated friction coefficients, see Table 1. In all cases the following was defined.

• 4% friction coefficient on all lubricated contact surfaces
• Initial contact between lever caps and BB i.e., 0 mm gap
• 0.5 mm gap on the conical contact surfaces between housing and BB

An increase of the friction coefficient between lever cap and lever increases the moment that can be transferred from the lever cap to the lever and hence the bending stresses in the level, see Fig. 11.

## 4 Conclusion

The GI is the component of the BB transporter with the highest force density. The structural integrity assessment showed it feasible to transfer the huge moments due to the off-centered position of the BB segment during the lifting as well as the high payload. Given the absence in EN13001 of rules for a limit analysis, we adopted the corresponding rules defined in RCC-MRx [18] to verify that the high stresses in local regions of the GI were not membrane stresses and will not cause excessive deformation. The collapse load factor was found to be 3.2, well above the required 1.5 according to the rules RCC MRx [18].

The GI is also capable to tolerate engagement gaps between the GI and the corresponding contact surfaces on the BB recess. The currently considered values of the engagement gap size is in line with standard manufacturing processes. Should the engagement procedure require larger gap sizes, these need to be verified in future structural assessments since the concept relies on the GI to tightly fit into the recess of the BB. The GI prevents in this way excessive deformation of the BB.

The friction coefficient on the dry contact surfaces was found to affect the bending stresses that occur in the levers. However, these remain within the stress limits even for a relatively high friction coefficient of 0.3, a typical value for steel-steel contact. Hence low friction surface coatings are not strictly required.

Our study neglected potential issues related to the reliability of the main components of the gripper since it is a pre-conceptual design. In this phase the authors paid attention in checking the structural integrity, the assembly tolerances and the potential friction coefficients defining also the areas that need to be lubricated. It is clear due to working environment the reliability of the main components of the GI is a critical aspects that needs to be addressed by future studies.

Further activities should be dedicated to the estimation of the sticking force between the cap lever surfaces and the corresponding on the BB segments, the reliability of the main components and actuation systems and about the alignment features (GI versus BB recess). A dedicated test campaign will be needed, also on scaled version of the gripper interlock, to address: the functionality of the gripper interlock, the sticking phenomenon and the reliability of the critical components (levers, cap levers, spherical pressure pads); the possibility of the system to tolerate small deformations on the working surfaces due to a repeated lifting operations of the BB segments.

## Author contribution statement

Rocco Mozzillo: Conceived and designed the experiments; Performed the experiments; Wrote the paper.

Christian Bachmann: Analyzed and interpreted the data; Contributed reagents, materials, analysis tools or data.

Pierluigi Fanelli: Contributed reagents, materials, analysis tools or data.

Guenter Janeschitz, Thomas Steinbacher: Conceived and designed the experiments.

## Data availability statement

Data included in article/supp. Material/referenced in article.

## Declaration of competing interest

The authors declare that they have no known competing financial interests or personal relationships that could have appeared to influence the work reported in this paper.
